# NADC30-Like Porcine Reproductive and Respiratory Syndrome in China

**DOI:** 10.2174/1874357901711010059

**Published:** 2017-06-30

**Authors:** Kegong Tian

**Affiliations:** 1 College of Animal Science and Veterinary Medicine, Henan Agricultural University, Zhengzhou, China; 2 OIE Porcine Reproductive and Respiratory Syndrome Laboratory, Beijing, China

**Keywords:** NADC30-like PRRSV, Recombination, Pathogenicity, Vaccine efficacy

## Abstract

NADC30-like porcine reproductive and respiratory syndrome virus (PRRSV) has widely spread in China and become locally dominant virus strain in some provinces. Although they are not pathogenic as highly pathogenic PRRSV (HP-RRRSV) that outbreaks since 2006, NADC30-like PRRSVs distinguished themselves by high incidence of recombination with other virus strains which lead to change of virulence. The outbreaks of NADC30-like PRRSV in the vaccinated pig herds suggested that current commercial PRRSV vaccines cannot provide complete protection to the infection. In this review, we have described in detail the current situation of NADC30 PRRSV including epidemiology, genomic characterization, pathogenicity, and efficacy of current commercial vaccines in China.

## INTRODUCTION

Porcine reproductive and respiratory syndrome (PRRS) is one of the most economically important infectious diseases of pigs worldwide [1]. The etiologic agent is porcine reproductive and respiratory syndrome virus (PRRSV) which belongs to the order *Nidovirales*, family *Arteriviridae* [2]. PRRSV can be divided into European genotype 1 and North American genotype 2 with Lelystad and VR-2332 as prototypical strains, respectively [3]. PRRSV is featured by mutation and recombination which lead to the emergence of new field strains with different virulence [4, 5]. Several studies have reported the change of pathogenicity and virulence due to the virus genome mutations or recombination [4, 6-10].

Since 2014, several research groups have reported the field isolates of PRRSV in South China with a very unique genetic background [11, 12]. These viruses shared the highest nucleotide similarity to a group represented by NADC30, a type 2 PRRSV that was previously reported in Unites States of America in 2008 [13]. In the past two years, NADC30-like PRRSV has spread to several provinces and become the dominant strain locally in the vaccinated pigs [11, 12]. Compared to the highly pathogenic PRRSV (HP-PRRSV) which has emerged and become the dominant circulating strain in China since 2006, the virulence of NADC30-like PRRSV is relative milder which has led to clinical respiratory symptoms with 30-50% fatality rate on pigs [12, 14, 15]. The outbreak of NADC30-like PRRSV in vaccinated pig herds indicated the inefficacy of current commercial vaccines [12]. In this review, we have described the genomic characteristics and pathogenicity of NADC30-like PRRSV as well as the efficacy of current commercial vaccines to NADC30-like PRRSV.

## GENERAL SITUATION OF PRRSV IN CHINA

North American genotype (genotype 2) PRRSV was first reported in China in 1995 [16]. After that, PRRS has become endemic in pig herds nationwide [17, 18]. These PRRSV strains were classified as classical PRRSV (C-PRRSV) that clinically characterized by reproductive failure of sows and decreased growth performance and increased mortality in weaning pigs [19, 20]. The significance of economic loss due to classical PRRS at that time was not well documented until the outbreak of HP-PRRSV in 2006. European genotype (genotype 1) PRRSV was firstly reported in China in 2011 [21]. Two European genotype PRRSV BJEU06-1 and NMEU09-1 shared 91.5% and 87.0% identity with Lelystad virus. The genome identity between these two PRRSVs was only 84.9% which indicated they were probably from different ancestors. Later on, four genotype 1 PRRSV isolates NVDC-NM1-2011, NVDC-NM2, NVDC-NM3, and NVDC-FJ were reported in 2015 [22]. They shared 87.4%-90.7% identity with Lelystad PRRSV and 85.6%-95.5% with each other. However, the pathogenicity of above European genotype of PRRSV was never reported. The emergence of European genotype of PRRSV since last decade makes PRRSV control in China as more difficult and challenging.

The first case of HP-PRRSV was reported in Jiangxi province of China in 2006 [23]. The infection of pigs of all ages featured by high fever (41-42°C), high morbidity (50%-100%) and mortality (20%-100%) was initially addressed as “swine high fever disease (SHFD)” [24, 25]. In the next couple of months, the disease spread to most provinces of China and some other Asian countries such as Mongolia, Vietnam, Thailand, India, and Laos [26-30]. The outbreak of HP-PRRSV resulted in a dramatic decline of pig stock and high prices of pork meat in China [31]. Tian group firstly identified HP-PRRSV as the etiological agent to SHFD and successfully established infection model in SPF pigs with PRRSV isolate JXA1 [23]. Compared to classical PRRSV, pig infected with HP-PRRSV exhibits more severe clinical symptoms including respiratory distress, conjunctivitis, diarrhea, and neural signs with high morbidity and mortality [32]. At autopsy, more dramatic pulmonary edema and consolidation, bleeding and necrosis of lymphoid tissues and severe lesions in brain were observed. At the same time, HP-PRRSV has broader tissue tropism that could extend to liver, kidney, brain, and heart besides lung and lymphoid tissues [33]. Merely in 2006, the outbreak of HP-PRRSV in China led to the death of more than 1 million pigs and had a huge economic losses to Chinese pig industry [23]. After that, HP-PRRSV became the dominant circulating PRRSV strain in China.

Since 2013, several outbreaks of PRRSV in the vaccinated pigs have been reported in Central China such as Henan province [11]. The infected pregnant sows had abortions and stillbirth and the weaning pigs had clinical respiratory disorders [12]. These PRRSV isolates had a very unique genetic background and showed the highest nucleotide similarity to a group represented by NADC30, a type 2 PRRSV that has been isolated in United States of America in 2008 [12]. Therefore, these viruses were designated as NADC30-like PRRSV in China. Compared to the above-mentioned classical PRRSV and HP-PRRSV, NADC30-like PRRSV has very unique genomic characters and distinct pathogenicity [8]. The detailed information will be presented below.

## GENOMIC CHARACTERISTICS OF NADC30-LIKE PRRSV

So far, six NADC30-like PRRSV genomes were released and became available in Genbank Table (**1**). All NADC30-like PRRSV have 131-aa discontinuous deletions in the nonstructural protein 2 (nsp2) including 111-aa deletion at position 322-432, 1-aa deletion at position 483, and a 19-aa deletion at position 504-522 which could distinguish themselves from other PRRSV strains Fig. (**1**). On genomic level, NADC30-like PRRSV shared 93.8%-95.6% nucleotide similarity to NADC30, 87.1%-89.8% to VR-2332 (prototype of classical type 2 PRRSV), and 82.3%-90.7% to JXA1 (prototype of HP-PRRV). Among the six reported NADC30-like PRRSV, they shared 88.4%-97.8% nucleotide similarity with each other.

A phylogenetic analysis of above six NADC30-like PRRSVs with other published PRRSV strains was conducted by using a distance-based neighbor-joining method with 1,000 bootstrap replicates in MEGA6. As shown in Fig. (**2**), these six PRRSV isolates were shown to be genetically more closely related to NADC30 and clustered into a separate branch (cluster 3). All HP-PRRSV field isolates and vaccine strains formed another cluster represented by JXA1 and HuN4-F114 respectively (cluster 1). Meanwhile, all classical genotype 2 PRRSV vaccine strains and field isolates represented by Ingelvac MLV and Ch-1a were clustered in a separate branch (cluster 2). The low similarity between NADC30-like PRRSVs with current widely used PRRSV vaccine strains may indicate the incomplete protection provided by vaccination.

## High incidence of recombination in NADC30-like PRRSVs

As a RNA virus, PRRSV featured by high rates of mutation may work as a strategy that virus may utilize to escape the host immune surveillance [34]. Unlike other PRRSV strains, NADC30-like PRRSVs distinguished themselves by high incidence of recombination Table (**2**). Among six reported NADC30-like PRRSV, five viruses were found to recombine with other PRRSV strains including both classical PRRSV and HP-PRRSV. According to PRRSV strains (classical PRRSV strains vs HP-PRRSV strains) they recombined, these NADC30-like PRRSV strains could be divided into three groups. The first group represented by HNjz15 (Genbank Access No. KT945017.1) has the highest similarity with NADC30 PRRSV and no recombination with other viruses. Group 2 represented by Chsx1401 (KP861625.1) has the recombination with classical PRRSV including VR-2332 and CH-1a. PRRSV in group 2 includes Chsx1401, HENA-XINX (KF611905.1), and HNyc15 (KT945018.1). Group 3 represented by JL580 (KR706343.1) has the recombination with HP-PRRSV such as 09HEN1. In this group, HENAN-HEB (KJ143621.1) has the recombination with HP-PRRSV JXA1 strain. As shown in Table (**2**), the hotspots for recombination of NADC30-like PRRSV with other virus strains locate in both nonstructural protein regions including nsp1a, nsp2-9, nsp 11 and structure protein region such as ORF2-5.

An interesting phenomena for above recombination is that the recombination could occur between parental NADC30 strain with either classical or HP-PRRSV. An example is that NADC30-like PRRSV strain HNyc15 has the recombination with classical PRRSV strains VR-2332 and CH-1a. However, there was no recombination event with both classical and HP-PRRSV observed so far.

Different patterns of recombination with some virus strains lead to distinct cell tropism of NADC30-like PRRSV [8, 11]. Among above mentioned six NADC30-like PRRSV, only JL580 was reported to be proliferated on Marc145 cells [8]. The other five viruses could only be propagated in porcine alveolar macrophage (PAM) but not in Marc145 cells. The genome analysis of JL580 reveals the recombination with HP-PRRSV 09HEN1 occurring in nsp2, nsp3, nsp7, ORF2a, and ORF4. ORF2-4 was reported to be associated with cell tropism and HP-PRRSV 09HEN1 can be propagated in Marc145 cells with high virus titer which could partially explain the distinct cell tropism of JL580 as compared with other five NADC30-like PRRSV strains [35].

## PATHOGENICITY OF NADC30-LIKE PRRSV

NADC30 was originally isolated from Iowa herds experiencing outbreaks of respiratory disease in 2008 [13]. Compared to other two PRRSVs (MN184 and SDSU73) performed in the same study, pigs infected with NADC30 had an early febrile and peak at day 2 and day 8 post-infection. Pig body temperature above 40°C occurred on day 1-3, 6, and 8-11. Consistent with the body temperature, NADC30-infected pigs also developed earlier viremia which could be detected as early as day 1 post-infection. The NADC30-infected pigs developed interstitial pneumonia, but relatively milder as compared with SDSU73 and MN184. No pigs were found dead by the end of the study. The above results suggested NADC30 PRRSV as relatively mild in virulence.

Consistent with NADC30, HNjz15, a NADC30-like PRRSV without any recombination with other PRRSVs showed much lower pathogenicity as compared with HP-PRRSV [36]. Sun, *et al.* compared pathogenicity between HNjz15 and JXA1 on 6-week old pigs with same virus titer for inoculation. Similar to NADC30, pigs infected with HNjz15 developed fever at day 1 post-infection and lasted for consecutive 9 days. Clinically, HNjz15-infected pigs suffer from respiratory distress such as dyspnea, tachypnea and coughing. Serum virus RNA copy numbers in HNjz15-infected pigs were significantly lower than that in JXA1-infected pigs throughout the study. All HNjz15-infected pigs survived by the end of study (terminated at 14 dpi). In contrast, two pigs were found dead in JXA1-infected group. Histopathologically, interstitial pneumonia associated with hemorrhage was observed in HNjz15-infected pigs. Interstitial pneumonia was characterized by thickening of alveolar septa and infiltration of mononuclear cells. Lymphoid tissues such as tonsil and lymph nodes also exhibited lymphocyte depletion, acute hemorrhage and infiltration of neutrophils. But the total histopathological parameters in JXA1-infected group were significantly higher than HNjz15- infected group. The low proliferation ability, limited tropism of PRRSV HNjz15, and mild clinical symptoms of pigs induced by HNjz15 infection showed that it was not pathogenic as HP-PRRSV JXA1.

However, NADC30-like PRRSV JL580 is highly pathogenic to 6-week pigs with 100% mortality within two weeks [8]. In Zhao’s study, both cell culture-originated JL580 (2^nd^ passage in Marc145 cells) and lung homogenate supernatant of virus were used for pig inoculation. The infected pig showed high fever from 3 day post-inoculation (dpi) and had obvious clinical manifestations including cough, anorexia, blue ears and discolor of body. The infected pigs started to die at 7 dpi and all pigs were dead by the end of the study (terminated at 14 dpi). The high pathogenicity of JL580 may explained by the fact of discontinuous recombination with HP-PRRSV 09HEN1 and some exchanged gene fragments may happen to be the virulence-determined genes.

## EFFICACY OF CURRENT COMMERCIAL VACCINES TO NADC30-LIKE PRRSV

The outbreak of NADC30-like PRRSV indicates inefficacy of current commercial PRRSV vaccines. So far, five commercial PRRSV vaccines by using their corresponding virus strains including JAXA1-P80, HuN4-F112, GDr180, TJM-F92, and VR-2332 are widely used in China. The efficacy of these PRRSV vaccines to HNjz15 infection was tested on 6-week old pigs (unpublished data). The vaccinated pigs in each individual group were challenged at 28 days post vaccination. Compared to unvaccinated pigs, the vaccinated pigs clinically shortened the period of fever and fewer pigs showed clinical symptoms and had improved body weight gain. After HNjz15 challenge, the pigs in all vaccinated groups had the similar level of viremia as the unvaccinated groups which indicated vaccination could not effectively remove the circulating virus in pigs. Also, the virus load in tonsil, lung and lymph nodes detected by immunohistochemistry staining in vaccinated pigs was similar to that in unvaccinated pigs. Therefore, the above results suggested that current commercial PRRSV vaccines could not provide complete protection to the NADC30-like PRRSV infection.

## CONCLUSION

Since its first outbreak in 2013, researchers have paid high attention to NADC30-like PRRSV due to its high incidence of recombination with other virus strains. Even though the parental NADC30 (including HNjz15 NADC30-like PRRSV without recombination) has limited pathogenicity to pigs, the risks of its recombination with other virus strains which lead to the emergence of highly pathogenic PRRSV such as JL150 will severely endanger local pig industry. The high pathogenicity of JL150 was presumably due to the discontinuous large gene fragments of recombination spanning its genome between parental NADC30 strains with HP-PRRSV 019HNE [37]. However, the above assumption needs to be addressed to make such conclusion by testing other NADC30-like PRRSV isolates which have the similar patterns of recombination with classical PRRSV strains. By using the well-established reverse genetic techniques on PRRSV, the above question should be addressed in the near future [38].

The inefficacy of current PRRSV vaccines to the circulating NADC30-like PRRSV in the field or experimental infection may lead the pig herds into dangers. Even though some NADC30-like PRRSVs were mildly virulent to pigs, the deceased body weight gain and co-infection with other swine pathogens due to NADC30-like PRRSV infection could also cause huge economic losses to Chinese pig industry. Therefore, more measurements including development of NADC30-like PRRSV vaccine should be put into consideration to control the spreading of this disease.

## SUPPLEMENTARY MATERIAL

Supplementary material is available on the publishers Website along with the published article.

## Figures and Tables

**Fig. (1):**
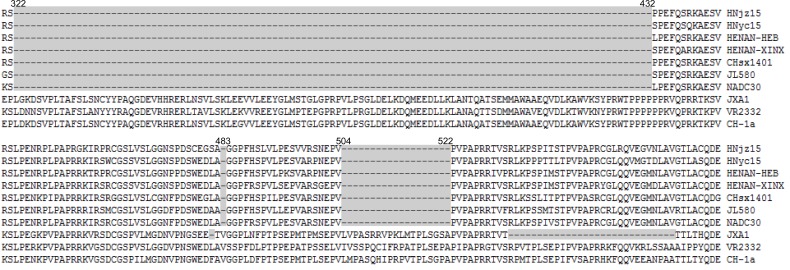
The discontinuous deletions in nonstructural protein 2 (nsp2) of NADC30-like PRRSVs

**Fig. (2):**
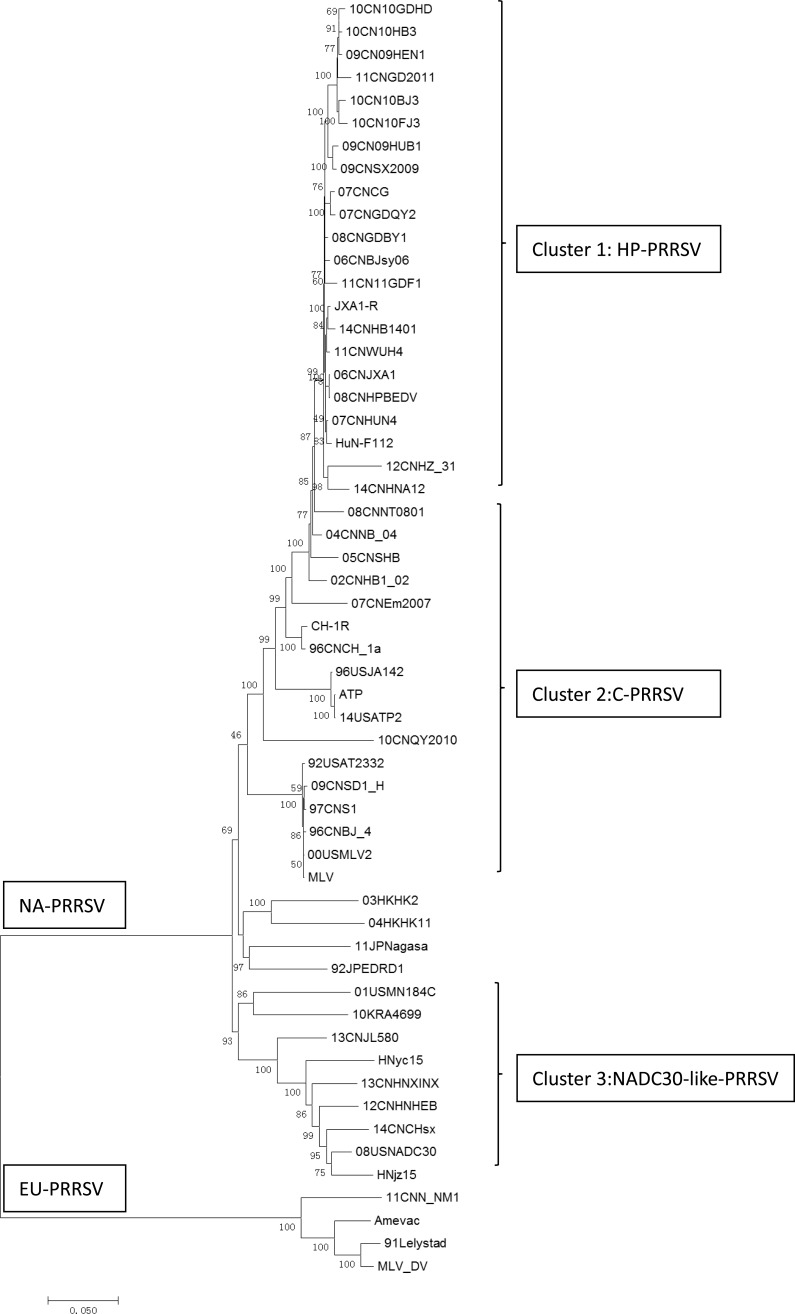
Phylogenetic analysis of whole genomes of six NADC30-like PRRSVs with other 54 PRRSVs (10 vaccine strains and 44 field isolates). Phylogenetic analysis was performed by using a distance-based neighbor-joining method with 1,000 bootstrap replicates in MEGA6. Numbers along branches are bootstrap values. Scale bar indicates nucleotide substitute per site.

**Table 1:** Information of six NADC30-like PRRSVs in this review.

**Virus**	**Genbank Acesss No.**	**Isolation year**	**Isolation site**
HNjz15	KT945017.1	2015	Henan province
HNyc15	KT945018.1	2015	Henan province
HENAN-HEB	KJ143621.1	2013	Henan province
HENAN-XINX	KF611905.1	2013	Henan province
JL580	KR706343.1	2014	Jilin province
Chsx1401	KP861625.1	2015	Shanxi province

**Table 2:** Recombination sites of six NADC30-like PRRSVs in this review.

**Virus**	**Recombination ** **strain **	**Recombination ** **with**	**Recombination** ** sites**
HNjz15	/	/	/
HNyc15	VR-2332/CH1a	Classical PRRSV	ORF2-4
HENAN-HEB	JXA1	HP-PRRSV	NSP2
HENAN-XINX	VR-2332	Classical PRRSV	Nsp2-5
JL580	09NEN1	HP-PRRSV	Nsp2、Nsp3、Nsp7、ORF2a、ORF4
Chsx1401	VR-2332	HP-PRRSV	Nsp11

## References

[1] Lunney J.K., Fang Y., Ladinig A. (2016). Porcine Reproductive and Respiratory Syndrome Virus (PRRSV): Pathogenesis and Interaction with the Immune System.. Annu. Rev. Anim. Biosci..

[2] Loving C.L., Osorio F.A., Murtaugh M.P., Zuckermann F.A. (2015). Innate and adaptive immunity against Porcine Reproductive and Respiratory Syndrome Virus.. Vet. Immunol. Immunopathol..

[3] Chae C. (2016). Porcine respiratory disease complex: Interaction of vaccination and porcine circovirus type 2, porcine reproductive and respiratory syndrome virus, and Mycoplasma hyopneumoniae.. Vet. J..

[4] Kappes M.A., Faaberg K.S. (2015). PRRSV structure, replication and recombination: Origin of phenotype and genotype diversity.. Virology.

[5] Brar M.S., Shi M., Murtaugh M.P., Leung F.C. (2015). Evolutionary diversification of type 2 porcine reproductive and respiratory syndrome virus.. J. Gen. Virol..

[6] Chen N., Yu X., Wang L. (2013). Two natural recombinant highly pathogenic porcine reproductive and respiratory syndrome viruses with different pathogenicities.. Virus Genes.

[7] Yu X., Chen N., Wang L. (2012). New genomic characteristics of highly pathogenic porcine reproductive and respiratory syndrome viruses do not lead to significant changes in pathogenicity.. Vet. Microbiol..

[8] Zhao K., Ye C., Chang X.B. (2015). Importation and Recombination Are Responsible for the Latest Emergence of Highly Pathogenic Porcine Reproductive and Respiratory Syndrome Virus in China.. J. Virol..

[9] Li Y., Ji G., Wang J., Tan F., Zhuang J., Li X. (2016). Complete Genome Sequence of an NADC30-Like Porcine Reproductive and Respiratory Syndrome Virus Characterized by Recombination with Other Strains.. Genome Announc..

[10] Yuan S., Nelsen C.J., Murtaugh M.P., Schmitt B.J., Faaberg K.S. (1999). Recombination between North American strains of porcine reproductive and respiratory syndrome virus.. Virus Res..

[11] Li C., Zhuang J., Wang J. (2016). Outbreak Investigation of NADC30-Like PRRSV in South-East China.. Transbound. Emerg. Dis..

[12] Zhou L., Wang Z., Ding Y., Ge X., Guo X., Yang H. (2015). NADC30-like Strain of Porcine Reproductive and Respiratory Syndrome Virus, China.. Emerg. Infect. Dis..

[13] Brockmeier S.L., Loving C.L., Vorwald A.C., Kehrli M.E., Baker R.B., Nicholson T.L. (2012). Genomic sequence and virulence comparison of four Type 2 porcine reproductive and respiratory syndrome virus strains.. Virus Res..

[14] Jiang P., Chen P.Y., Dong Y.Y., Cai J.L., Cai B.X., Jiang Z.H. (2000). Isolation and genome characterization of porcine reproductive and respiratory syndrome virus in P.R. China. Journal of veterinary diagnostic investigation: official publication of the American Association of Veterinary Laboratory Diagnosticians.. Inc..

[15] Li L., Zhao Q., Ge X. (2012). Chinese highly pathogenic porcine reproductive and respiratory syndrome virus exhibits more extensive tissue tropism for pigs.. Virol. J..

[16] Guo B.Q., Chen Z.S., Liu W.X., Cui Y.Z. (1996). Isolation and Identification of porcine reproductive and respiratory syndrome (PRRS) virus from aborted fetuses suspected of PRRS.. Chin J. Anim. Poult Infect. Dis..

[17] Liu C, Ning Y, Xu B, Gong W, Zhang D (2016). Analysis of genetic variation of porcine reproductive and respiratory syndrome virus (PRRSV) isolates in Central China.. The Journal of veterinary medical science / the Japanese Society of Veterinary Science.

[18] Zhang Q., Xu X., You S. (2016). Emerging of two new subgenotypes of porcine reproductive and respiratory syndrome viruses in Southeast China.. Microb. Pathog..

[19] Li H., Yang H. (2003). Infection of porcine reproductive and respiratory syndrome virus suppresses the antibody response to classical swine fever virus vaccination.. Vet. Microbiol..

[20] Jiang P., Jiang W., Li Y., Wu S., Xu J. (2004). Humoral immune response induced by oral administration of S. typhimurium containing a DNA vaccine against porcine reproductive and respiratory syndrome virus.. Vet. Immunol. Immunopathol..

[21] Chen N., Cao Z., Yu X. (2011). Emergence of novel European genotype porcine reproductive and respiratory syndrome virus in mainland China.. J. Gen. Virol..

[22] Zhou Z., Liu Q., Hu D. (2015). Complete genomic characterization and genetic diversity of four European genotype porcine reproductive and respiratory syndrome virus isolates from China in 2011.. Virus Genes.

[23] Tian K., Yu X., Zhao T. (2007). Emergence of fatal PRRSV variants: unparalleled outbreaks of atypical PRRS in China and molecular dissection of the unique hallmark.. PLoS One.

[24] Yu X., Chen N., Deng X. (2013). Genomic sequencing reveals mutations potentially related to the overattenuation of a highly pathogenic porcine reproductive and respiratory syndrome virus.. Clin. Vaccine Immunol..

[25] Zhou Z., Ni J., Cao Z. (2011). The epidemic status and genetic diversity of 14 highly pathogenic porcine reproductive and respiratory syndrome virus (HP-PRRSV) isolates from China in 2009.. Vet. Microbiol..

[26] Ni J., Yang S., Bounlom D. (2012). Emergence and pathogenicity of highly pathogenic Porcine reproductive and respiratory syndrome virus in Vientiane, Lao People's Democratic Republic. Journal of veterinary diagnostic investigation: official publication of the American Association of Veterinary Laboratory Diagnosticians.. Inc..

[27] Jantafong T., Sangtong P., Saenglub W., Mungkundar C., Romlamduan N., Lekchareonsuk C. (2015). Genetic diversity of porcine reproductive and respiratory syndrome virus in Thailand and Southeast Asia from 2008 to 2013.. Vet. Microbiol..

[28] Rajkhowa T.K., Jagan Mohanarao G., Gogoi A., Hauhnar L., Isaac L. (2015). Porcine reproductive and respiratory syndrome virus (PRRSV) from the first outbreak of India shows close relationship with the highly pathogenic variant of China.. Vet. Q..

[29] Chaikhumwang P, Tantituvanont A, Tripipat T, Tipsombatboon P, Piriyapongsa J, Nilubol D (2015). Dynamics and evolution of highly pathogenic porcine reproductive and respiratory syndrome virus following its introduction into a herd concurrently infected with both types 1 and 2. 2015;30:164-74.. Infection, genetics and evolution : journal of molecular epidemiology and evolutionary genetics in infectious diseases..

[30] Nguyen V.G., Kim H.K., Moon H.J. (2015). Evolutionary Dynamics of a Highly Pathogenic Type 2 Porcine Reproductive and Respiratory Syndrome Virus: Analyses of Envelope Protein-Coding Genes.. Transbound. Emerg. Dis..

[31] Feng Y., Zhao T., Nguyen T. (2008). Porcine respiratory and reproductive syndrome virus variants, Vietnam and China, 2007.. Emerg. Infect. Dis..

[32] Wang G., Yu Y., Tu Y. (2015). Characterizing the thymic lesions in piglets infected with attenuated strains of highly pathogenic porcine reproductive and respiratory syndrome virus.. Vet. Immunol. Immunopathol..

[33] Wang G., Yu Y., Tu Y. (2015). Highly Pathogenic Porcine Reproductive and Respiratory Syndrome Virus Infection Induced Apoptosis and Autophagy in Thymi of Infected Piglets.. PLoS One.

[34] Lyoo Y.S. (2015). Porcine reproductive and respiratory syndrome virus vaccine does not fit in classical vaccinology.. Clin. Exp. Vaccine Res..

[35] Tian D., Wei Z., Zevenhoven-Dobbe J.C. (2012). Arterivirus minor envelope proteins are a major determinant of viral tropism in cell culture.. J. Virol..

[36] Sun Z., Wang J., Bai X. (2016). Pathogenicity comparison between highly pathogenic and NADC30-like porcine reproductive and respiratory syndrome virus.. Arch. Virol..

[37] Zhang Q., Yoo D. (2015). PRRS virus receptors and their role for pathogenesis.. Vet. Microbiol..

[38] Han M., Yoo D. (2014). Engineering the PRRS virus genome: updates and perspectives.. Vet. Microbiol..

