# Supplementary material for: NADC30-Like Porcine Reproductive and Respiratory Syndrome in China
Source: Open Virol J. 2017 Jun 30;11:59–65. doi: 10.2174/1874357901711010059 (PMC5543618; doi:10.2174/1874357901711010059)
Supplement: Supplementary file 1 [file TOVJ-11-59_SD1.pdf]

Send Orders for Reprints to reprints@benthamscience.ae

The Open Virology Journal, 2017, 11, i-ii

BENTHAM OPEN

CrossMark

The Open Virology Journal

Supplementary Material

Content list available at: www.benthamopen.com/TOVJ/

DOI: 10.2174/1874357901711010059

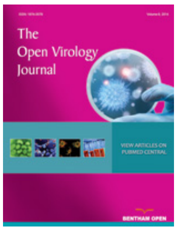

NADC30-Like Porcine Reproductive and Respiratory Syndrome in China

Kegong Tian[1,2,*]

[1]College of Animal Science and Veterinary Medicine, Henan Agricultural University, Zhengzhou, China
[2]OIE Porcine Reproductive and Respiratory Syndrome Laboratory, Beijing, China

Received: October 06, 2016 Revised: November 01, 2016 Accepted: February 06, 2017

SUPPLEMENTAL DATA

Suppl 1. Information of PRRSV strains used in phylogenetic analysis. Vaccine strains were indicated by the names in bold.

Virus strain	Isolation Year	Country	Access No.
10CN10GDHD	2010	China	JX215553
10CN10HB3	2010	China	JQ663553
09CN09HEN1	2009	China	JF268684
11CNGD2011	2011	China	KC527830
10CN10BJ3	2010	China	JQ663542
10CN10FJ3	2010	China	JQ663548
09CN09HUB1	2009	China	JF268682
09CNSX2009	2009	China	FJ895329
07CNCG	2007	China	EU864231
07CNGDQY2	2007	China	GU454850
08CNGDBY1	2008	China	GQ374442
06CNBJsy06	2006	China	EU097707
11CN11GDF1	2011	China	JX215551
**JXA1-R**	2009	China	FJ548853
14CNHB1401	2014	China	KM261784
11CNWUH4	2011	China	JQ326271
06CNJXA1	2006	China	EF112445
08CNHPBEDV	2008	China	EU236259
07CNHUN4	2007	China	EF635006
**Hun4-F112**	2010	China	/
12CNHZ_31	2012	China	KC445138
14CNHNA12	2014	China	KJ819934
08CNNT0801	2008	China	HQ315836
04CNNB_04	2004	China	FJ536165
05CNSHB	2005	China	EU864232
02CNHB1_02	2002	China	AY150312
07CNEm2007	2007	China	EU262603
**CH1R**	2008	China	EU807840
96CNCH_1a	1996	China	AY032626

Suppl 1 contd.....

Virus strain	Isolation Year	Country	Access No.
96USJA142	1996	USA	AY424271
**ATP**	2006	USA	DQ988080
**14USATP2**	2014	USA	EF532801
10CNQY2010	2010	China	JQ743666
92USAT2332	1992	USA	U87392
**09CNSD1_H**	2009	China	GQ914997
97CNS1	1997	China	DQ459471
96CNBJ_4	1996	China	AF331831
**00USMLV2**	2000	USA	AF159149
**MLV**	1998	USA	AF066183
11JPNagasa	2011	Japan	AB811786
92JPEDRD1	1992	Japan	AB288356
03HKHK2	2003	HongKong	KF287133
04HKHK11	2004	HongKong	KF287138
10KRA4699	2010	Korea	JX138236
01USMN184C	2001	USA	EF488739
15HNyc	2015	China	KT945018
13CNHNXINX	2013	China	KF611905
12CNHNHEB	2012	China	KJ143621
08USNADC30	2008	USA	JN654459
15HNjz	2015	China	KT945017
11CNN_NM1	2011	China	JX187609
**Amevac**	2009	Spain	GU067771
91Lelystad	1991	Netherland	A26843
**MLV-DV**	1999	Netherland	KJ127878

© 2017 Kegong Tian.

This is an open access article distributed under the terms of the Creative Commons Attribution 4.0 International Public License (CC-BY 4.0), a copy of which is available at: https://creativecommons.org/licenses/by/4.0/legalcode. This license permits unrestricted use, distribution, and reproduction in any medium, provided the original author and source are credited.
